# Polymer-Based Nanomaterials and Applications for Vaccines and Drugs

**DOI:** 10.3390/polym10010031

**Published:** 2018-01-02

**Authors:** Jinyu Han, Dandan Zhao, Dan Li, Xiaohua Wang, Zheng Jin, Kai Zhao

**Affiliations:** 1 Key Laboratory of Chemical Engineering Process and Technology for High-efficiency Conversion, College of Chemistry and Material Sciences, Heilongjiang University, Harbin 150080, China; hanjinyu1018@126.com; 2 Key Laboratory of Microbiology, School of Life Science, Heilongjiang University, Harbin 150080, China; zhaodandan@hlju.edu.cn (D.Z.); docor1005@163.com (D.L.); zybin395@163.com (X.W.); 3 School of Biological Science and Technology, University of Jinan, Jinan 250022, China

**Keywords:** vaccines, drugs, polymer-based nanomaterials, nanoparticles, delivery carriers, vaccine adjuvants

## Abstract

Nanotechnology plays a significant role in drug development. As carriers, polymeric nanoparticles can deliver vaccine antigens, proteins, and drugs to the desired site of action. Polymeric nanoparticles with lower cytotoxicity can protect the delivered antigens or drugs from degradation under unfavorable conditions via a mucosal administration route; further, the uptake of nanoparticles by antigen-presenting cells can increase and induce potent immune responses. Additionally, nanomaterials are widely used in vaccine delivery systems because nanomaterials can make the vaccine antigen long-acting. This review focuses on some biodegradable polymer materials such as natural polymeric nanomaterials, chemically synthesized polymer materials, and biosynthesized polymeric materials, and points out the advantages and the direction of research on degradable polymeric materials. The application and future perspectives of polymeric materials as delivery carriers and vaccine adjuvants in the field of drugs and vaccines are presented. With the increase of knowledge and fundamental understandings of polymer-based nanomaterials, means of integrating some other attractive properties, such as slow release, target delivery, and alternative administration methods and delivery pathways are feasible. Polymer-based nanomaterials have great potential for the development of novel vaccines and drug systems for certain needs, including single-dose and needle-free deliveries of vaccine antigens and drugs in the future.

## 1. Introduction

Vaccination is considered one of the greatest breakthroughs in modern medicine. As the most effective treatment method ever developed [1,2,3], vaccines are a weapon that humans can use to control or eliminate a disease, ranging from human immunodeficiency virus (HIV) [4,5] to hepatitis C [6]. Many lives have been saved, and quality of life has been improved through the use of vaccines, including for cancer and virus-infected patients [7,8,9]. In recent years, new vaccines are developed by using modern biotechnology, including recombinant subunit vaccines, synthetic peptide vaccines, DNA vaccines, and nano vaccines [10,11,12,13]. With the development of modern design concepts, immune system enhancers or drug delivery systems are used to improve immunogenicity, minimize toxicity, and enhance effectiveness [14]. Immune system enhancers can enhance the specific immune response, and can therefore be used as a vaccine adjuvant [15]. Vaccine adjuvants include immune stimulants, carriers, and nanoadjuvants. The majority of nanomaterials belong to the latter two types of adjuvants. As non-viral vectors, nanomaterials have several advantages, including good biocompatibility, mucosal adsorption, and biodegradability. Other unique physicochemical properties of nanomaterials as non-viral vectors include an ease of processing and modification, controllable surface properties, the promotion of functional molecules into cells, and the protection of DNA and proteins from degradation. Therefore, nanomaterials research is a hot spot in the field of vaccine adjuvants and drug delivery carriers [16,17].

The chemical and physical properties of nanomaterials have attracted the attention of scientists, and nanomaterials have been widely explored in the field of drug development and delivery. Many nanomaterials have been developed, including iron oxide nanoparticles, gold nanoparticles, cerium oxide nanoparticles, carbon-based nanomaterials, and polymeric nanoparticles. Nanomaterials have exhibited great potential in the applications of vaccines and drugs. Additionally, nanomaterials themselves have antigenic or medicinal activity, and can elicit a strong immune response. Nanomaterials can inhibit or even kill pathogenic cells by using their cellular toxicity or autoimmunity [18]. At present, some nanomaterials are used as vaccine adjuvants and delivery carriers, antibacterial and antitumor drugs, or as drug delivery carriers. As shown in Table 1, polymer-based nanomaterials are among the nanomaterials with high biological safety and good biodegradability, which can protect the antigens or drugs from degradation [19]. Polymer-based nanomaterials include chitosan, polylactic acid (PLA), polyglutamic acid (PGA), poly(lactic-glycolic acid) (PLGA), etc.

Polymer-based, nano-controlled release systems are solid colloidal particles with a particle size of 10–500 nm, and bioactive materials that are internalized through dissolving, wrapping, or adsorption and adhesion on the surface of the particles. Polymer-based nanomaterials have also become a new vehicle for the controlled release of drugs. The main difference between nanoparticle carriers and microparticle carriers is the ultrasmall volume of the former. Stability, chemical structure, and particle size can be artificially controlled through chemical means to control the rate of drug or gene release. The introduction of recognizable molecules on the surface of the particle gives it the ability to recognize target cells, and also permits the gradual degradation of the polymer within the cell. It is therefore widely used in drug development and delivery [20,21].

In the present review, we have reviewed and discussed recent reports of the applications of polymer-based nanomaterials in the production of vaccines and drugs. The review focuses on polymer-based nanomaterials as vaccine adjuvants and delivery systems for vaccines and drugs. This review will help to understand the advantages of natural, chemically synthesized, and biosynthesized polymeric materials that are used as vaccine adjuvants or delivery systems in the biomedical field.

## 2. Polymeric Nanomaterials

### 2.1. Natural Polymer-Based Nanomaterials

Natural polymer is a renewable resource that can be obtained from a variety of sources; it can be degraded into water, carbon dioxide and inorganic small molecules; it can also be classified as an environmentally-friendly material. Natural polymers with a variety of functional groups can be produced by physical and chemical methods, or through modification to become a new material using emerging nanotechnology. At present, commonly used natural polymer materials include chitosan, starch, alginate, cellulose, hyaluronic acid, chondroitin sulfate, etc.

**Figure 1:**
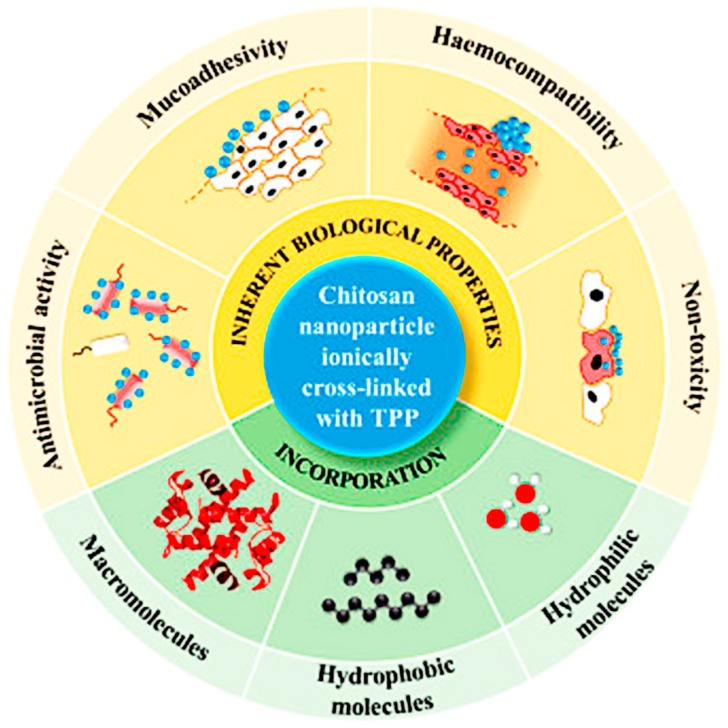
Characteristics of chitosan/triphenyl phosphate nanoparticles considered in this review: their inherent biological properties and ability to incorporate many bioactive species within them. Reprinted from Reference [54].

Chitosan has good biocompatibility and biodegradability, and can bind to a negatively-charged protein or plasmid DNA through electrostatic interaction to form polymer composites that protect the protein and DNA from degradation [22], and is therefore suitable as an adjuvant or delivery carrier. In addition, chitosan has good adsorbability, permeability, and moisture retention. The abundant amino and hydroxyl groups on the chitosan skeleton make it amenable to chemical modification. Chitosan derivatives with certain functional groups can be obtained by the chemical modification of chitosan. However, the performance of derivatives is often better than chitosan [53]. There are many studies on chitosan derivatives obtained through such processes as acylation, alkylation, sulfation, hydroxylation, quaternization, and carboxymethylation [23,24,25,26,27]. Among them, products obtained through quaternization and carboxymethylation have received increasing amounts of attention because of their good water solubility. Due to their significant inherent biological properties, including their antimicrobial activity and non-toxicity, chitosan derivatives have immense potential in the biomedical field (Figure 1) [54].

Starch widely exists in grains, beans, and potato crops; it is non-toxic, has no immunogenicity, has good storage stability, does not interact with drugs, and is therefore widely used in the pharmaceutical industry. Starch is used as a drug carrier, as tablet excipients, in bone repair, and as a protective agent in plasma and frozen blood cells. Starch microspheres, after reaching micron and nanoscale levels, exhibit the following surface and volume effect. The microsphere surface area increases, the concentration of functional groups and selective adsorption capacity increases, the adsorption equilibrium time shortens, and the colloid stability and slow-release effect both become more prominent [28,29,30]. 

Alginate is a natural polysaccharide that is obtained mainly from marine plants, namely *Chlorophyceae*, *Cyanophyceae*, *Phaeophyceae,* and *Rhodophyceae* [31,32]. At present, the widely used alginate products that are commercially available include sodium alginate, calcium alginate, and ammonium alginate [33]. Alginates are classified into low viscosity, moderate viscosity, and high viscosity, according to the composition of alginate and relative molecular mass. The variability in the performance of alginates derives from the functional diversity of the material. Some of the beneficial functional properties of alginates include good moisture absorption, easy removal, high oxygen permeability, gelation obstructive, biodegradability, biocompatibility, and the adsorption of metal ions, which make them very useful in the medical field [34,35].

### 2.2. Biosynthesized Polymer Materials

Biosynthesized polymers are obtained through enzyme hydrolysis (using microbial enzymes). These compounds contain microbial polyesters and microbial polysaccharides. Representative products are poly-β-hydroxybutyrate (PHB), poly(3-hydroxybutyrate-*co*-3-hydroxyvalerate), biofiber bundle, polyamino acid, etc.

PHB is a kind of high molecular polymer produced by microorganisms under unfavorable growth conditions (when nutrients become limited) [36]. An environmentally friendly and novel solvent system to recover the PHB from *Cupriavidus necator* has been used, and PHB recovery of 83% and purity of 90% were achieved [37]. PHB has properties (biodegradability, biocompatibility, piezoelectricity, optical activity, and other special properties) akin to synthetic polymers [38]. This high molecular polymer can not only be applied as a drug delivery carrier, it can also be used in tissue engineering as a scaffold material, and in surgical treatment as a bone repair material. In vitro and in vivo testing showed that insulin-loaded deoxycholic acid that had been conjugated with PEGylated polyhydroxybutyrate co-polymeric nanoparticles increased the intestinal absorption of insulin to provide a sustained hypoglycemic effect beyond 24 h [39]. PHB are widely used in suture surgery because there is no need for after surgery removal, as it can degrade in vivo. PHB is also used for soft tissue repair (such as skin and palatal tissue repair), and also in wound support material, vascular substitutes, and blood bags, because of the ability of PHB to desorpt on serum protein and fiber protein [51,55].

### 2.3. Chemically Synthesized Polymer Materials

Chemically synthesized polymer materials, including PLA, PLGA, polyurethane (PU), poly(methyl methacrylate), polyester, polyvinylpyrrolidone (PVP), silicone rubber, polyvinyl alcohol, etc., that are used in medical materials are produced through chemical methods.

PLA and its copolymer are biocompatible and biodegradable, and can be obtained from a wide range of raw material sources. They are renewable, non-toxic, and completely biodegradable, and recognized by the Food and Drug Administration (FDA). They have good mechanical strength, elastic modulus, and thermal formability, and are used in bone tissue engineering, cartilage regeneration, cartilage repair, and as a carrier in the formulation of controlled-release drugs [40]. When used as a carrier in sustained-release drugs, PLA helps release the drug gradually through its slow degradation in vivo. PLA and its copolymers have short half-lives, poor stability, and an ease of degradation. Their use as carriers in the formulation of controlled-release drugs makes it easier to effectively widen the dosage, reduce the dosing frequency and dosage [41], enhance the effective drug concentration, and minimize the side effects of drugs on the body, especially on the liver and kidney [42].

PU materials have good compatibility (biological, blood, and tissue compatibility), excellent fatigue resistance, wear resistance, high elasticity and high strength when compared with other polymer materials. PU materials are therefore widely used in the field of biomedical materials, such as in the production of artificial organs, catheter interventions, and polymeric drug capsules [43]. The main performances of PU include excellent clotting, low toxicity, non-teratogenic, non-allergic, and non-carcinogenic [46]. With the continuous expansion of PU applications in the field of medical biology, PU has the disadvantage that it can’t be naturally degraded, and will ultimately lead to environmental pollution; this situation is increasingly becoming an obstacle to the development of polyurethane. Therefore, the development of biodegradable PU materials becomes the key to solving this problem. Currently available biodegradable PU include oligosaccharides-derived PU; lignin, tannin, and bark-derived PU; cellulose derivatives of PU; and starch derivatives of PU [44,47]. Since PU has good biological compatibility and resists thrombosis, biodegradable PU has great development potential in the biomedical field.

PLGA is a kind of biodegradable polymer with good biocompatibility and degradability, and is widely used in microspheres, microcapsules, nanoparticles, pellets, implants, and film preparation. Drugs encapsulated into PLGA nanoparticles or microspheres increase in the tumor site, and drugs administered in PLGA nanoparticles or microspheres reduce the adverse reaction; they also prolong the cycle time of chemotherapy drugs in vivo, and improve the pharmacokinetic properties of drugs [45,48,49,50]. As a new type of drug controlled-release material, PLGA copolymers have been widely used at home and abroad in the controlled release of chemotherapeutic agents, antibiotics, peptides, proteins, polysaccharides, and other drugs. The factors affecting the physical properties and drug release of microspheres have also been studied. In a recent study, a biodegradable PLGA-based microparticle that was loaded with a bacterial-produced prodigiosin drug obtained from *Serratia marcescens* subsp, caused the controlled release of prodigiosin for 30 d, and the release reduced the cell viability of MDA-MB-231 cells [56]. Polyoxyethylene (PEG) as a delivery material for drugs is often used to modify the system in the form of PLGA nanoparticles or microspheres. The hydrogen bonding between the core materials of hydroxyl or amino groups and PEG chain oxygen atoms can improve the encapsulation efficiency [51,57]. Jogala et al. prepared and evaluated intravenous sustained-release stealth nanoparticles encapsulating low molecular weight heparin (LMWH) using different grades of PEG and PLGA [58]. Using a Water/Oil/Water (W/O/W) solvent evaporation technique helped successfully develop an effective stealth LMWH nanoparticle formulation for the treatment of venous thrombosis. These results provided a reliable basis for optimizing the preparation process of microspheres for the purpose of obtaining good sustained-release properties [59,60,61].

## 3. Application of Polymer-Based Nanomaterials

### 3.1. Polymer-Based Nanomaterials as a Delivery Carrier for Vaccines

Although vaccines are the most effective treatment method ever developed, most vaccines are still administered via injection as a result of the lack of an appropriate delivery system to reach the induction position and enhance the immune responses. The mucosal route has great potential, since application through mucosa can stimulate both mucosal and systemic immunity. Mucosal vaccines can prevent the entrance of the pathogens into the body, the inactivation of pathogens, and the dissemination of pathogens [62]. Additionally, mucosal vaccines also have lots of advantages over injectable vaccines by being simpler to administer, having less risk of transmitting infections, and potentially being easier to manufacture [63]. However, mucosal vaccines are not without problems. Some of the problems associated with mucosal vaccines are limited humoral and cell-mediated responses, a low delivery of potentially protective viral epitopes, and inefficiencies of adjuvants used in different protocols [64]. Developing mucosal vaccines based on biomaterial nanoparticles as adjuvants and delivery carriers can circumvent some of the shortcomings of conventional vaccines. The nanovaccines can enhance humoral, cell-mediated, and mucosal immune response through sustained release, and also protect loaded antigen against degradation.

Nanoscale carriers can be divided into organic and inorganic material carriers. Inorganic material carriers have some disadvantages, such as lack of immunogenicity and lack of cell targeting, and can’t be degraded in vivo. The organic material carrier has more application prospects because they are relatively safe, non-toxic, non-immunogenic, and non-carcinogenic. Most of the traditional organic carriers are cationic polymers and liposome derivatives. The biggest defect is that the gene delivery efficiency is low, especially in vivo, because of easy digestion by intracellular hydrolase, and lack of tissue specificity [65]. The ideal delivery carrier should have the characteristics of high delivery efficiency, low cytotoxicity, no side effect on normal cell physiology, an ease of application, and good reproducibility. As a delivery vehicle, the main advantages of polymer-based nanomaterials are as follows: it is a non-viral vector, is non-immunogenic, has good biocompatibility, and has a large specific surface area. They are easy to load with model drugs and deliver to specific tissues, organs, or cells. These specific advantages would greatly benefit the administration of vaccines in both human and veterinary medicine.

**Figure 2:**
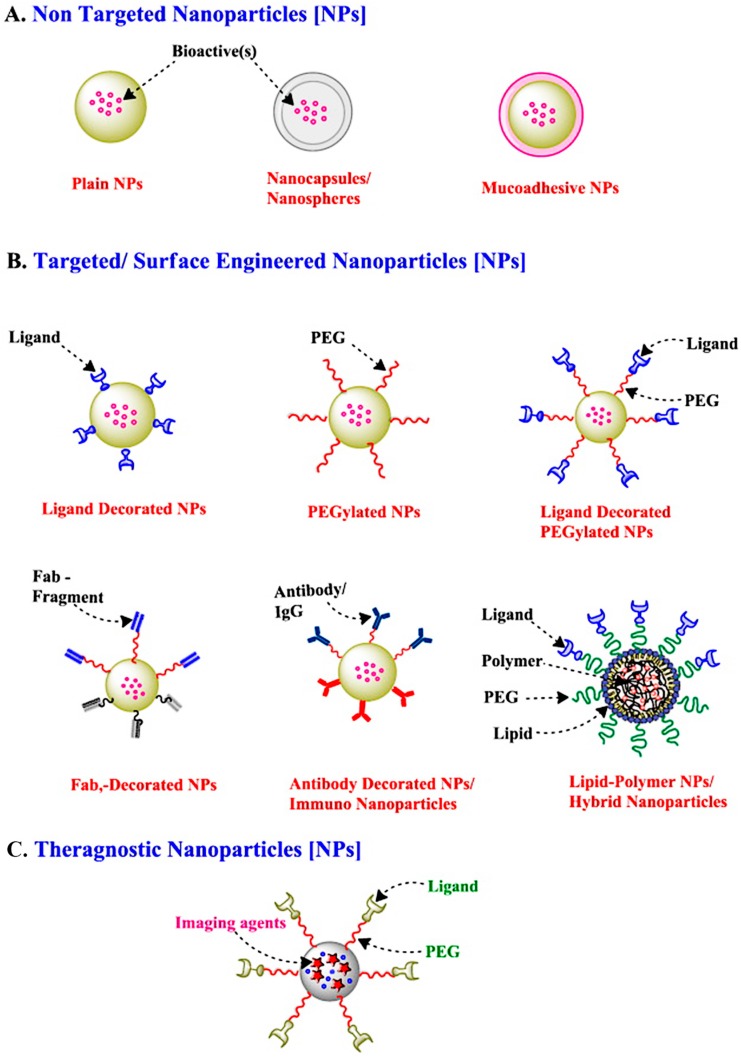
Various types of nanoparticles as mucosal vaccines delivery candidates. Reprinted from Reference [63].

At present, in order to obtain desired vaccine delivery carriers, several natural or synthetic polymers are used as fundamental materials for the preparation of nanoparticles (Figure 2) [63]. Due to an increase of active constituent absorption under the nanoparticles, the therapeutic dose can be significantly reduced compared with traditional drugs. Polymer nanomaterials that are biodegradable also play a vital role in the polymeric nanoparticles’ metabolic process in vivo.

There is considerable excitement within the nanotechnology field for nanosystems as carriers for vaccines and drugs. As a delivery vector, nanomaterials have many advantages. Our group developed Newcastle disease virus (NDV) encapsulated in *N*-2-hydroxypropyl trimethyl ammonium chloride chitosan nanoparticles (NDV/La Sota-N-2-HACC-NPs), and found the NDV/La Sota-N-2-HACC-NPs to have very low toxicity, a high level of safety, and stronger cellular, humoral, and mucosal immune responses than commercial vaccines; it also exhibited sustained release [66]. The hydrophilic microgel of starch is sensitive to salt ions, pH, temperature, storage time, and environmental changes, which can lead to different degrees of swelling and shrinkage. Microgel is expected to be developed as a new type of drug carrier. The better biocompatibility of starch materials and their adhesion to living cells prevent the degradation of drugs and assure the controlled release of the drug.

### 3.2. Polymer-Based Nanomaterials as Vaccine Adjuvants

Various immunogens that are used in vaccine formulations need adjuvants to elicit the immune response. With traditional adjuvants, which include various kinds of aluminum derivatives and emulsions, the degree of dispersion depends on the particle size distribution of the inner-phase droplets, which may be changed during preparation or after injection in the body. For instance, the droplet size may increase after injection into fatty tissues, and cause the emulsion droplets to coalesce into larger particles. Presently, aluminum hydroxide adjuvants are preferred for use in humans and animals for safety reasons. In addition, aluminum-based adjuvants are the only adjuvants currently included in vaccines licensed by the FDA. Aluminum adjuvants selectively stimulate a Th2 immune response upon the injection of mice, and activate the CD8 T cells. The adsorption of antigens to aluminum adjuvants enhances the immune response by facilitating phagocytosis [67]. Hogenesch et al. evaluated the preclinical safety of the recombinant *Streptococcus pyogenes* vaccine formulated with aluminum adjuvant, and the data showed that repeat dose toxicity did not lead to significant vaccine-associated adverse effects in outbred CD-1 mice [68].

**Figure 3:**
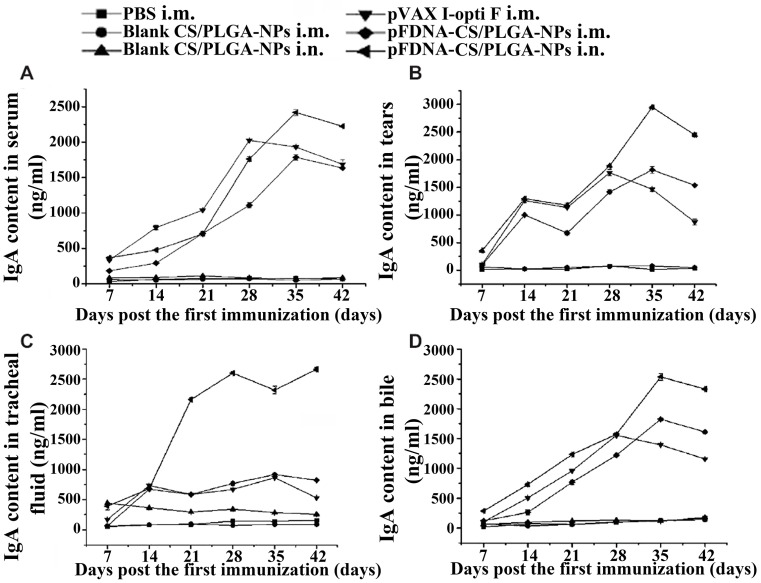
Immunoglobulin A (IgA) antibody content in serum (**A**); tears (**B**); tracheal fluid (**C**); and bile (**D**) of specific pathogen free (SPF) chickens immunized with PBS i.m., blank CS/PLGA NPs i.m., blank CS/PLGA NPs i.n., pVAX I-optiF i.m., pFDNA-CS/PLGA-NPs i.m., and pFDNA-CS/PLGA-NPs i.n. Values are means ± SD (*n* = 5). Reprinted from Reference [69].

The advantage of polymeric particles is that they can be prepared from renewable materials, and their particle size and constituent can be controlled. Nanoparticles include micron and nanopolymers that are used as carrier particles for vaccines or drugs. Drugs or vaccine antigens could be covered with particles by polymerization. Alternatively, drugs or antigens could also be bound to the nanoparticles by adsorption to the surface of the nanoparticles by chemical bonding. Our group has prepared chitosan-coated poly(lactic-glycolic acid) (PLGA) nanoparticles containing the eukaryotic expression plasmid DNA of NDV F gene (pVAX I-optiF). The nanoparticles could control the burst release of plasmid DNA, and induced stronger cellular, mucosal, and humoral immune responses than the naked plasmid DNA vaccine alone (Figure 3). The results indicated that chitosan-coated PLGA nanoparticles can serve as an efficient and safe mucosal immune delivery system for the NDV DNA vaccine [69].

### 3.3. Nanoparticle-Based Drug Delivery System

Nanoparticle-based drug delivery systems are nanoscale carriers that are used to deliver drugs or vaccines. In brief, nanoparticles also include sub-micro particles with sizes below 1000 nm, such as nanocapsules, nanomicelles, and nanoliposomes [70]. 

Nanoparticle-based drug delivery systems have some advantages: as a result of their ultrasmall volume, these systems can cross the smallest blood capillary and avoid elimination by phagocytes, thus prolonging their duration in the bloodstream. They can easily pass through tissue and gaps because of their volume, and reach target organs such as the spinal cord, lung, spleen, lymph, and liver. They also have controlled-release properties on account of their biodegradability, temperature sensitivity, and pH. Due to these advantages, they can reduce toxic side effects, and improve the utility of drugs, and are therefore widely used in drug formulations. 

As drug delivery systems, nanoparticles can absorb drugs or vaccines onto their exterior surfaces, or entrap drugs or vaccines into their interior structures. Presently, nanoparticles are widely used in many areas, including the delivery of polypeptides [39], proteins [60], vaccines [15], nucleic acids [53], and genes [48]. Over the years, nanoparticle-based drug delivery systems have exhibited huge potential in medical, biological, and pharmaceutical applications [71].

Research work on nanoparticle-based drug delivery systems include the surface modification of nanoparticles to increase target characteristics; the selection of carrier materials to get suitable drug release speeds; and optimizing the preparation of nanoparticles to improve their drug delivery capability. Research also seeks to explain the in vivo interaction of nanoparticles with targeted tissues, organs, and blood [72]. Of course, polymeric materials applied to prepare nanoparticles for drug delivery should be biodegradable and biocompatible. For this purpose, lots of polymeric materials have been widely used, including polysaccharides [67], PLA [40], proteins [73], or polypeptides [74]. In some ways, polysaccharides such as chitosan are the most widely applied polymeric materials for drug delivery in the field of nanomedicine.

Polysaccharide-based nanoparticles have received attention from many scholars [75]. Over time, more and more polysaccharide-based nanoparticles will be available with diverse functionality. Based on their structural characteristics, nanoparticles are able to form ionic and covalent cross-linkages, as well as self-assemble and form polyelectrolyte complexes with hydrophobically-modified polysaccharides. 

### 3.4. Antibacterial Application of Polymer-Based Nanomaterials

Antibacterial agents are compounds that slow down the growth or partly kill bacteria without harm to surrounding tissues. Currently, most antibacterial agents are chemically modified natural compounds [76], such as β-lactams (such as penicillins), carbapenems, or cephalosporins. Of course, there are some pure natural products, such as aminoglycosides, and also purely synthetic antibiotics, such as sulfonamides, for use as antibacterial agents. Generally, these agents can be defined as either bacteriostatic (slow down bacterial growth) or bactericidal (kill bacteria). Antibacterial agents are important for the prevention and control of communicable diseases. However, the abuse of antibacterial agents has led to antibiotic resistance. Antibiotic resistance has become a global concern, as it is most often the cause of evolutionary processes taking place during antibiotic therapy. Moreover, horizontal gene transfer by transformation, transduction, and conjugation can be a way to enhance resistance [77]. Such antibacterial-resistant strains are informally regarded as superbugs, and contribute to the emergence of diseases that are difficult to treat. One classic example is bacterial strains causing tuberculosis that is resistant to current treatment methods. Out of the estimated 480,000 cases of multidrug-resistant tuberculosis in 2013, only 136,000 were reported to the World Health Organization (WHO) [78]. 

In addition, traditional antimicrobial agents do not only cause adverse reactions, they also cause multiple drug resistance. In order to fight drug resistance, the dose of antibiotics is often increased, which leads to intolerable toxicity. This has led to the development of alternative strategies to treat bacterial disease [79]. Among them, nanoscale materials have emerged as novel antimicrobial agents. It is a remarkable fact that several classes of nanoscale carriers and antimicrobial nanoparticles for antibiotics delivery have indicated their effectiveness for treating communicable disease [80]. Nanoparticles serve as better antibacterial agents, because they have a high surface area to volume ratio, and unique mechanical, electro-optical, chemical, magnetic, electrical, magneto-optical, and optical properties [81]. Nanoparticles have been demonstrated as effective in the environment against bacteria.

AgNPs-chitosan nanoparticles were more efficient in inhibiting the growth of *E. coli* compared with either AgNPs or chitosan. The negatively charged surface of the cells attached to the positively charged chitosan to enhance the binding of AgNPs to proteins that were present on the cell walls [82]. This is believed to lead to intracellular constituents and other leakage of proteins, and therefore deactivation of the bacteria. The addition of AgNPs to chitosan was also used to prevent the formation of biofilms specific to *E. coli* and the purification of water.

## 4. Future Perspectives

Biodegradable polymer-based nanomaterials offer suitable applications in the drug carrier and biomedical fields. A wide range of biodegradable polymer-based nanomaterials has been exploited in the preparation of biodegradable nanoparticles for the delivery of loaded model drugs in required dosages to specific areas of the body. The prominent advantages of biodegradable polymer nanoparticles are their biocompatibility and biodegradability in different delivery systems. The preparation of nanoparticles as drug delivery systems uses different techniques. Herein, we have attempted to present some of the different aspects of the work that has already been conducted. There is still much work to be done for nanoparticles to achieve wide usage. We believe that more nanoparticle drug delivery systems will become available in the near future. Currently, research work on nanoparticles is restricted to their physicochemical properties and toxicity, including: the specific interaction of nanoparticles with the cells, tissues, organs, and biomolecules in the body; the effect on the body’s metabolism; and their use as antibacterial agents.

## Figures and Tables

**Table 1:** Classification, application areas, advantages, and disadvantages of polymer-based nanomaterials.

Classification	Materials	Application Areas	Advantages	Disadvantages
Natural polymeric material	Chitosan	Hemostasis material, medical dressing, hydrogel, drug delivery carrier, gene transfer [22]	Biocompatibility, antimicrobial, innocuous, easily degradable, adsorbability, film formation [23,24,25,26,27]	Poor spinnability, poor strength, low water-solubility [23,25]
	Starch	Hemostasis material, tissue-engineered scaffold, drug delivery carrier, bone repair material [28,29]	Extensive sources, low price, degradation products safe and non-toxic, non-antigenic [28,30]	Poor mechanical properties, resistance to water, poor blocking performance [28,30]
	Alginate	Pharmaceutical excipient, pepcid complete, medical dressing [31,32,33,34,35]	Hypotoxicity, biocompatibility, suppresses tumor growth, enhances immunity [31,32,33,34,35]	Bad biodegradability, cell attachment poor [31,32,33,34,35]
	Cellulose	Pharmaceutical adjuvant [28]	Extensive sources, low price [28]	Rare adverse reactions [28]
Biosynthesis material	Poly β-hydroxybutyrate (PHB)	Drug-delivery carrier, tissue engineering material [36,37,38]	Biodegradable, safe, non-toxic, good physical and chemical properties [37,39]	High crystallinity, bad thermal stability [37,39]
Chemosynthes material (Copolymer)	Polylactic (PLA)	Anti-adhesion materials, patch, drug-delivery carrier, bone-fixing device, suture, tissue-engineered scaffold [40,41,42]	Biocompatibility, good mechanical properties, safe, non-toxic [40,41,42]	Poor toughness, degradation speed slow, hydrophobicity, lack of reactive side chain groups [40,41,42]
	Polyurethane	Excipients, medical bandage [43,44,45]	Low cost, rich resource, good mechanical properties [43,44,46,47]	Degradation speed slow [43,46,47]
	Poly(lactic-glycolic acid) (PLGA)	Absorbable suture, drug delivery, bone screw fixation, tissue repair [48,49,50,51]	Controllable biodegradability, biocompatibility [48,49,50]	Higher cost, drug-loading capacity and stability can be improved [48,49,50]
	Polymethyl methacrylate resin (PMMA)	Bone-fixation materials, dental materials, artificial crystal [52]	Easy operation, good biocompatibility	Monomer has cytotoxicity, easy oxidation

## Abbreviations

PBS: phosphate buffered saline; CS: chitosan; i.m.: intramuscular; i.n.: intranasal; pVAX I-optiF: eukaryotic expression plasmid DNA of Newcastle disease virus F gene; pFDNA-CS/PLGA-NPs: chitosan-coated pVAX I-optiF encapsulated in PLGA nanoparticles.
